# Description of Streptomyces borealis sp. nov. and Streptomyces saintjeani sp. nov., associated with potato common scab

**DOI:** 10.1099/ijsem.0.007254

**Published:** 2026-07-27

**Authors:** Adrien Biessy, Anuradha U. Jayathissa, Martin Filion

**Affiliations:** 1 Department of Plant Science, McGill University, Sainte-Anne-de-Bellevue, Quebec, Canada

**Keywords:** genome-based taxonomy, multilocus sequence analysis, polyphasic taxonomy, potato common scab, *Streptomyces *sp. nov.

## Abstract

Six *Streptomyces* strains were isolated from scab-infected potato tubers collected in the province of Quebec, Canada. All isolates produced disease symptoms characteristic of potato common scab. Multilocus sequence analysis showed that the strains clustered into two putative novel species-level groups represented by strains LBUM1542^T^ and LBUM3076^T^. Strains LBUM1542^T^ and LBUM3076^T^ shared the highest 16S rRNA gene sequence similarity with *Streptomyces rishiriensis* NBRC 13407^T^ (99.73%) and *Streptomyces acidiscabies* ATCC 49003^T^ (99.67%), respectively. Genome-based taxonomic analyses, including digital DNA–DNA hybridization and average nucleotide identity, supported the delineation of two novel species. Comparative genomic analyses revealed that both strains lacked the thaxtomin biosynthetic gene cluster classically associated with potato common scab but possessed other virulence determinants. Their predicted carbohydrate-active enzyme repertoires also differed markedly from those of their closest relatives. Morphological and phenotypic characteristics, together with genome-based chemotaxonomic predictions, were consistent with assignment to the genus *Streptomyces*. Some phenotypic traits, such as carbon source utilization, distinguished each strain from its closest phylogenetic neighbours. Taken together, these results demonstrate that LBUM1542^T^ and LBUM3076^T^ represent novel species, for which the names *Streptomyces borealis* sp. nov. (LBUM1542^T^=NCPPB 4834^T^=NRRL B-65796^T^) and *Streptomyces saintjeani* sp. nov. (LBUM3076^T^=NCPPB 4835^T^=NRRL B-65797^T^) are proposed.

## Introduction

Members of the genus *Streptomyces* are filamentous, spore-forming, Gram-positive bacteria that are ubiquitously found in soils, aquatic environments and both on and within plant and animal hosts [1, 2]. These micro-organisms are known for their complex life cycle [1] and for producing a tremendous diversity of secondary metabolites. Antibiotics produced by *Streptomyces* spp. have revolutionized human medicine by providing new weapons against ancient diseases [3, 4]. The search for novel microbial natural products continues to drive the discovery and formal description of many new *Streptomyces* species [5]. In 2025 alone, 66 novel *Streptomyces* species names were validly published according to the List of Prokaryotic names with Standing in Nomenclature [6, 7]. As a result of more than 80 years of continuous research, the genus *Streptomyces* is the largest prokaryotic genus, with more than 800 species with validly published and correct names [2, 6, 7].

Within this large genus, only a relatively small number of species are associated with plant diseases. Pathogenic *Streptomyces* spp. can infect various crop species, such as potato, sweet potato, carrot, radish, beet and turnip [8–10]. Among the diseases caused by these micro-organisms, potato common scab is the most economically significant [11]. Symptoms appear on progeny tubers and range from superficial corky lesions to deep-pitted or raised lesions [12]. Most species and strains causing this disease produce the phytotoxin thaxtomin A, although numerous additional virulence determinants have been characterized [13, 14]. *Streptomyces scabiei* (previously known as *S. scabies*) and *Streptomyces acidiscabies* were among the first scab-causing species to be described [15, 16]. These two species were described in North America and later found to be present in many potato-producing regions [17–19]. From 1996 to 2003, seven scab-causing *Streptomyces* species were formally described, namely *Streptomyces caviscabies*, *Streptomyces turgidiscabies*, *Streptomyces europaeiscabiei*, *Streptomyces stelliscabiei*, *Streptomyces luridiscabiei*, *Streptomyces puniciscabiei* and *Streptomyces niveiscabiei* [20–23]. No new scab-causing *Streptomyces* species were formally described for almost 20 years. However, in the past 4 years, six new species were formally described: *Streptomyces caniscabiei*, *Streptomyces griseiscabiei*, *Streptomyces hilarionis*, *Streptomyces hayashii*, *Streptomyces soliscabiei* and *Streptomyces echiniscabiei* [24–27]. The renewed interest of the scientific community in describing new scab-causing *Streptomyces* species reflects the threat this deleterious bacterial disease still represents to the potato crop more than three decades after the description of *S. scabiei*. This is also the direct consequence of the myriad of studies that surveyed the diversity of these micro-organisms in various potato-producing regions around the world, resulting in the isolation and characterization of thousands of strains [17, 19, 28–32].

In one of these studies, our team explored the diversity of *Streptomyces* species and strains causing potato common scab in the province of Quebec, Canada [29]. *S. scabiei* and *S. acidiscabies* were the most abundant scab-causing species found in the province, accounting for more than 80% of the strains isolated from common scab lesions [29]. Many strains were isolated from the Lac-Saint-Jean region, an area mostly dedicated to the production of certified seed tubers and where potato production is strictly regulated to prevent the introduction of new pathogens. Some of these strains were found to represent potential new species. In this study, we describe two novel *Streptomyces* species associated with potato common scab using a polyphasic approach that includes 16S rRNA phylogeny and multilocus sequence analysis (MLSA), genome-based taxonomy, genome mining for previously characterized virulence determinants, morphological and phenotypic characterization, carbohydrate-active enzyme profiling and genome-based prediction of chemotaxonomy.

## Methods

### Isolation of *Streptomyces* spp. from scab-infected potato tubers

*Streptomyces* strains were isolated from scab-infected potato tubers in 2020 and 2023 as previously described [29]. Briefly, diseased potato tubers were obtained from potato growers located in the Saguenay-Lac-Saint-Jean region (Quebec, Canada). Upon reception, the tubers were rinsed with water, left to dry for 1 day at ambient temperature and stored at 4 °C in paper bags. One scab lesion per potato tuber was gently scraped using a sterile scalpel and added to a 2-ml Lysing Matrix E tube (MP Biomedicals, Eschwege, Germany) filled with 1 ml of PBS containing 1% (vol/vol) Tween 80. The tissue was ground using a PowerLyzer 24 homogenizer (MO BIO, Carlsbad, CA) for 50 s at 2,800 r.p.m. The tube was then centrifuged for 1 min at 18,800 ***g***. The supernatant was serially diluted and plated on a semi-selective medium [33], prepared as described previously [28]. Petri dishes were incubated in the dark for 7 to 12 days at room temperature. Isolated colonies were purified by dilution-streaking on tryptic soy agar (BD Biosciences, San Jose, CA) and cryopreserved at −80 °C in tryptic soy broth (TSB) (BD Biosciences) supplemented with 20% (vol/vol) glycerol.

### Plant pathogenicity assay

The capacity of the strains under study to cause disease symptoms on potato tubers was evaluated using a pot experiment. Potato tubers were obtained from a local store and planted in 6.5-l plastic pots filled with a peat-based growing medium (PRO-MIX BX, Premier Tech, Rivière-du-Loup, QC, Canada). The tubers were inoculated with the *Streptomyces* strains on the same day by adding 100 ml of 2-week-old TSB-grown cultures. Plants were watered as required and fertilized every 2 weeks (starting on week 8) using a 10-15-10 fertilizer (Schultz liquid plant food, Sure-Gro IP, Brantford, ON, Canada). Potatoes were grown in growth chambers under a 16-h photoperiod (16 h of light at 23 °C followed by 8 h of darkness at 18 °C). After 9 weeks, the photoperiod was reduced to 10 h of light to favour tuber growth. Thirteen weeks after planting, potato progeny tubers were harvested, and the disease symptoms were observed. Four replicates per strain were used.

### Genomic DNA extraction

*Streptomyces* strains were grown in TSB for 7 days at 28 °C under continuous shaking, and genomic DNA was extracted using the DNeasy UltraClean Microbial Kit (QIAGEN, Toronto, Canada) following the manufacturer’s instructions. An additional incubation step at 70 °C was added prior to cell lysis to maximize yields. DNA yield and quality were evaluated using a NanoDrop 2000c spectrophotometer (Thermo Fisher Scientific, Waltham, MA). DNA samples were kept at −20 °C.

### Genome sequencing, assembly and annotation

The genomes of *Streptomyces* sp. LBUM1542 and LBUM1540 were sequenced in a previous study [29]. The DNA extracted from the four remaining *Streptomyces* strains under study was sent to the Integrated Microbiome Resource (Halifax, NS, Canada), where library preparation and genome sequencing were performed. Genomic DNA was mechanically sheared into fragments with a target size of 7 kb. These fragments were converted into SMRTbell libraries using the SMRTbell Express template prep kit v3.0 (Pacific Biosciences, Menlo Park, CA) as per the manufacturer’s instructions. Genome sequencing was carried out on a Sequel II sequencer (Pacific Biosciences) loaded with a SMRT Cell 8M (v2.0 chemistry). Genome assembly was performed using the long read assembler Flye v2.9.3-b1797 with default parameters [34]. The genomes were annotated by the (NCBI) National Center for Biotechnology InformationProkaryotic Genome Annotation Pipeline v6.10 [35].

### Virulence determinants associated with the development of potato common scab

Previously characterized genes and clusters associated with the development of the potato common scab disease were identified as described previously [29]. Briefly, the nucleotide sequences of numerous genes and clusters were extracted from reference genomes and used as baits in blast searches. The genome mining tool antiSMASH v8.0 was also used to confirm the presence and completeness of some of the biosynthetic gene clusters (BGCs) [36, 37].

### Phylogeny

To identify *Streptomyces* type strains that are closely related to the strains under study, multiple approaches were used. First, the 16S rRNA gene sequences were retrieved from the genomes of the six strains under study. These sequences were used as queries in blast searches on NCBI (limiting the search to sequences from type material). The EzBioCloud web server [38] was also used to identify closely related type strains. The 16S rDNA nucleotide sequences of *Streptomyces* type strains were retrieved from GenBank and aligned with clustal omega v1.2.2 [39]. The resulting alignment was used to generate an approximately maximum-likelihood tree with FastTree v2.1.11 [40, 41] and the generalized time-reversible (GTR) model. Secondly, we used a manually curated collection of more than 650 *Streptomyces* type strain genomes to generate an MLSA phylogeny. The complete nucleotide sequences of five housekeeping genes (*atpD*, *gyrB*, *recA*, *rpoB* and *trpB*) were extracted from the genomes and aligned using clustal omega. The resulting alignments were concatenated and used as an input to generate a phylogenetic tree with FastTree and the GTR model. A simpler phylogenetic tree was then generated using only a subset of strains. For all the trees generated in this study, the topology was verified by computing Shimodaira–Hasegawa support values [42], and *Actinomyces bovis* NCTC 11535^T^ was used as an outgroup. Finally, the Type (Strain) Genome Server was used to ensure that no closely related type strains were missed by the two approaches used. During the whole process, the List of Prokaryotic Names with Standing in Nomenclature website was regularly consulted to retain only type strains from species with validly published and correct names.

### Overall genome-relatedness indices

To determine whether the strains under study represent new species within the genus *Streptomyces*, three overall genome-relatedness indices (OGRIs) were calculated between the two representative strains and closely related type strains from previously described species. Digital DNA–DNA hybridization (dDDH) values were calculated with the Genome-to-Genome Distance Calculator v3.0 [43] using formula 2 (identities/HSP length). Average nucleotide identity based on blast+ (ANIb), average nucleotide identity based on MUMmer (ANIm) and tetra-nucleotide signature correlation indices were calculated using the JSpeciesWS web server [44].

### Morphology and physiology

One representative strain per potential new species (LBUM1542 and LBUM3076) was selected for further characterization. The strains were grown at 28 °C on various media, including yeast–malt extract (YME) agar, inorganic salts-starch (ISS) agar, oat bran agar and tryptone soy agar. The colour and aspect of the substrate mycelium, the aerial spore mass and the presence of diffusible pigments in the agar media were recorded. In addition, peptone–yeast extract iron (PYEI) agar and tyrosine agar were used to evaluate melanin production 4 days after inoculation. These media were prepared as described elsewhere [45, 46]. The ability of the strains to grow at a range of pH values (4–10), NaCl concentration (0–10%) and temperature (4, 10, 15, 20, 25, 28, 30, 37 and 42 °C) was evaluated in YME liquid medium. Carbon source utilization was evaluated on basal salt agar plates as described previously [45]. Phenotypic characterization, including Gram behaviour, oxidase/catalase activity, motility, nitrate reduction, gelatin liquefaction, starch hydrolysis and casein hydrolysis, was also performed. These analyses were carried out by DSMZ Services, Leibniz-Institut DSMZ – Deutsche Sammlung von Mikroorganismen und Zellkulturen GmbH (Braunschweig, Germany).

### Genome-based prediction of chemotaxonomy

To predict the presence/absence of several chemotaxonomic markers relevant for the description of new *Actinomycetota* species, we followed a method previously used in the formal description of several *Streptomyces* species [24, 27]. Amino acid sequences of enzymes involved in the biosynthesis of fatty acids, menaquinones, mycolic acids and 2,6-diaminopimelic acid were obtained from UniProt and used in blastp searches.

### Carbohydrate-active enzymes analysis

An *in silico* approach was used to compare the repertoire of carbohydrate-active enzymes (CAZymes) in the strains under study. Protein sequences were submitted to the dbCAN3 web server [47, 48], which annotated them using three different tools: HMMER:dbCAN, DIAMOND:CAZy and HMMER:dbCAN-sub. Only proteins with CAZy modules predicted by at least two tools were kept.

## Results and discussion

### Isolation, ecology, and plant pathology

Six *Streptomyces* strains were isolated from scab-infected potato tubers collected from various commercial potato fields located in Dolbeau-Mistassini (Saguenay-Lac-Saint-Jean region, Quebec, Canada). *Streptomyces* sp. LBUM1542 and *Streptomyces* sp. LBUM1540 were isolated in 2020 from common scab lesions on *S. tuberosum* ‘Colomba’. These two strains were described in a previous study [29]. Four strains (LBUM3076, LBUM3088, LBUM3223 and LBUM3237) were isolated in 2023 from common scab lesions on four distinct potato varieties (Table 1). All six strains were shown to produce common scab lesions on potato tubers (Fig. S1, available in the online Supplementary Material).

**Table 1.** Origin of the strains belonging to the two new species groups described in this study

Strain	Potato variety	Isolation date	Genome accession no.	Genome size (Mb)	G+C% (mol%)	No. contig
LBUM1542^T^	Colomba	2020	JARUNH000000000.1	9.87	71.4	3
LBUM1540	Colomba	2020	JARUMM000000000.1	9.89	71.3	6
LBUM3088	Upstate Abundance	2023	JBNHZZ000000000.1	10.02	71.3	7
LBUM3076^T^	W6511	2023	CP189624	9.83	71.0	1
LBUM3237	Chieftain	2023	JBNHUZ000000000.1	9.78	71.0	2
LBUM3223	Mountain Gem	2023	JBNHVL000000000.1	9.89	71.0	2

### Phylogeny and genome-based taxonomy

**Fig. 1.**
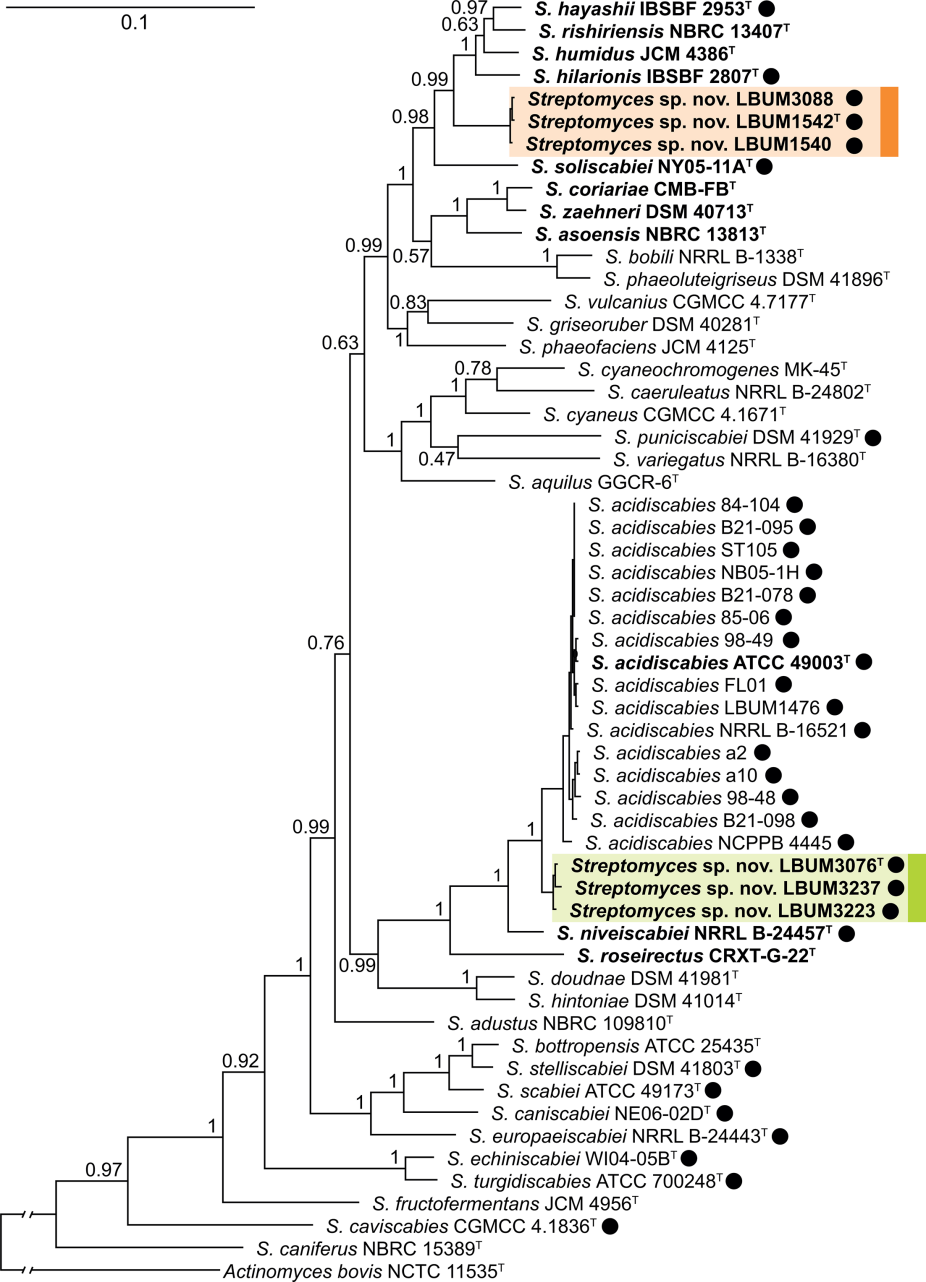
Multilocus sequence analysis based on the complete nucleotide sequences of five housekeeping genes (*atpD*, *gyrB*, *recA*, *rpoB* and *trpB*). This phylogenetic tree was generated using the approximate maximum-likelihood algorithm FastTree 2.1 and the GTR model. The topology was verified by computing Shimodaira–Hasegawa (SH) support values and *Actinomyces bovis* NCTC 11535^T^ was used as an outgroup. Support values within species groups are not displayed at the nodes. The scale bar indicates sequence divergence. The two new species groups described in this study are highlighted with colours. The six strains under study and the most closely related type strains are highlighted in bold. The presence of a black circle indicates phytopathogenic strains.

To visualize the phylogenetic placement of the six strains under study within the genus *Streptomyces*, a large-scale MLSA was carried out. The resulting maximum-likelihood tree, which includes more than 650 *Streptomyces* type strains, indicated that the six strains clustered into one clade within the genus *Streptomyces* (Fig. S2). To better visualize the phylogenetic relationships between the six strains under study and closely related type strains, a simpler MLSA phylogenetic tree was generated (Fig. 1). The strains clustered into two putative novel species-level groups represented by strains LBUM1542 and LBUM3076. Three strains (LBUM1542, LBUM3088 and LBUM1540) formed a group clearly separated from other *Streptomyces* type strains. The three other strains (LBUM3076, LBUM3237 and LBUM3223) clustered into one group close to *S. acidiscabies*. While these strains appear to be closely related to this scab-causing species, it is important to note that they formed a clearly delineated group separated from the various *S. acidiscabies* strains included in the tree (Fig. 1). A phylogenetic analysis was also conducted to identify closely related type strains based on 16S rRNA gene sequence similarity (Table S1 and Fig. S3). Strains LBUM1542 and LBUM3076 shared the highest 16S rRNA gene sequence similarity with *Streptomyces rishiriensis* NBRC 13407^T^ (99.73%) and *S. acidiscabies* ATCC 49003^T^ (99.67%), respectively. These analyses identified a total of 27 type strains as the closest relatives to the strains under study (Table S1). This includes the type strains of species associated with potato common scab, such as *S. acidiscabies*, *S. niveiscabiei*, *S. hayashii*, *S. hilarionis* and *S. soliscabiei* [16, 23, 26, 27].

To confirm whether these two groups represent novel *Streptomyces* species, several OGRIs were calculated between the two representative strains (LBUM1542 and LBUM3076) and the 27 most closely related type strains (Table S1). Digital DDH values calculated between *Streptomyces* sp. LBUM1542 and its closest related type strains ranged from 22.40 to 34.50%, which are well below the 70% threshold for species delineation. Likewise, the highest ANIb and ANIm values calculated were 86.62% and 89.31%, respectively, with *S. rishiriensis* NBRC 13407^T^. By comparison, *Streptomyces* sp. LBUM3076 was closely related to *S. acidiscabies*, as the dDDH value with the type strain of this species was equal to 63.00%. The ANIb and ANIm values with *S. acidiscabies* ATCC 49003^T^ were 94.94% and 95.70%, respectively. The ANIb value is lower than the threshold of 96% usually used for species delineation [49], and the ANIm value is well below the 96.7% recommended threshold for *Streptomyces* species delineation [50]. On the basis of these analyses, the six strains represent two new species groups. *Streptomyces* sp. nov. LBUM1542^T^ and *Streptomyces* sp. nov. LBUM3076^T^ were chosen as the proposed type strains.

### Genome features

**Fig. 2.**
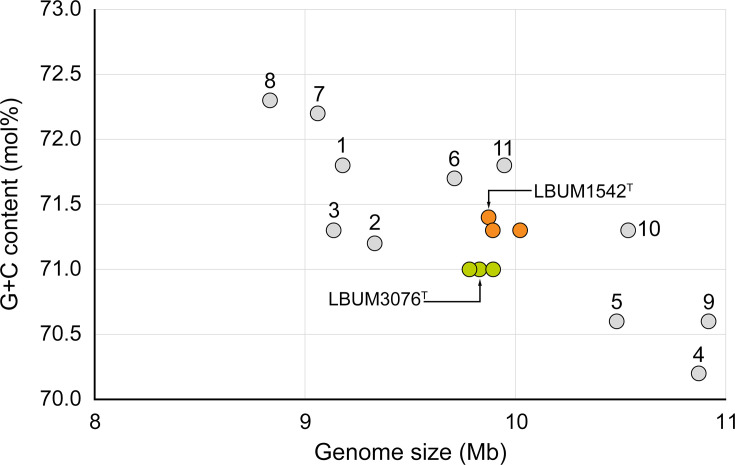
Genome size and G+C content for the *Streptomyces* strains under study and the most closely related *Streptomyces* type strains. Strains from the two novel species groups are represented as coloured circles, while closely related type strains are represented as grey circles. Numbered strains are as follows: 1, *S. rishiriensis* NBRC 13407^T^; 2, *S. coriariae* (CMB-FB^T^); 3, *S. zaehneri* (DSM 40713^T^); 4, *S. hayashii* IBSBF 2953^T^; 5, *S. soliscabiei* NY05-11A^T^; 6, *S. humidus* JCM 4386^T^; 7, *S. hilarionis* IBSBF 2807^T^; 8, *S. asoensis* NBRC 13813^T^; 9, *S. acidiscabies* ATCC 49003^T^; 10, *S. niveiscabiei* NRRL 24457^T^; and 11, *S. roseirectus* CRXT-G-22^T^.

The genomes of the six strains under study were sequenced using PacBio SMRT technology, yielding high-quality genome sequences (Table S2). The genome of *Streptomyces* sp. nov. LBUM1542^T^ was assembled into three contigs, with a total size of 9.87 Mb and a DNA G+C content of 71.4 mol%. The genome of *Streptomyces* sp. nov. LBUM3076^T^ was assembled into a single linear chromosome, with a length of 9.83 Mb and a G+C content of 71.0 mol%. These values fall within the range observed for the most closely related type strain genomes (Fig. 2). The genome of *Streptomyces* sp. nov. LBUM3076^T^ (9.83 Mb) was notably smaller than the genomes of *S. acidiscabies* ATCC 49003^T^ (10.92 Mb) and *S. niveiscabiei* NRRL 24457 ^T^ (10.54 Mb).

**Fig. 3.**
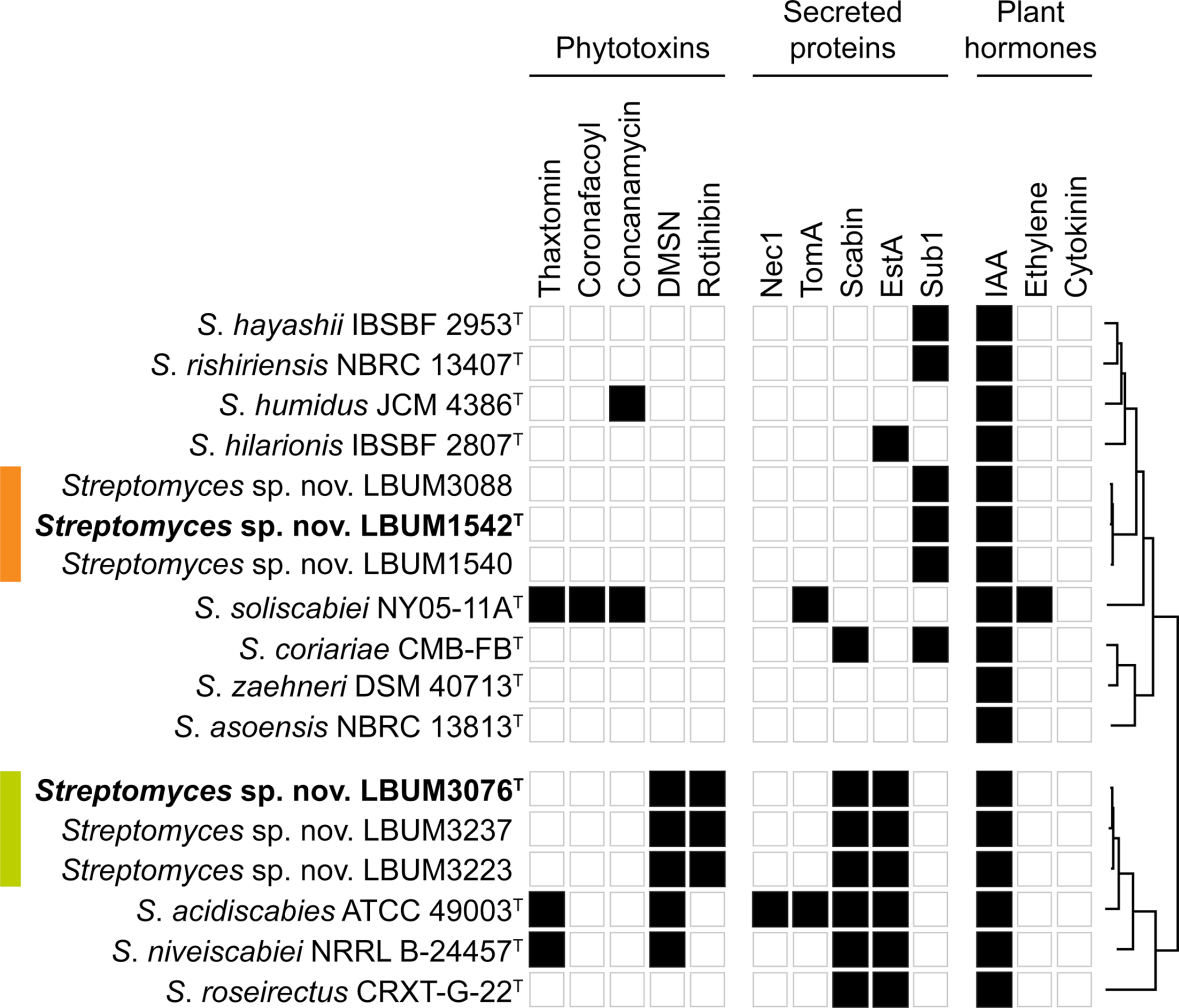
Distribution of previously characterized genes and clusters involved in the development of the common scab disease. The presence or absence of a gene/cluster in the genomes of the different strains is indicated by a square (black, presence; white, absence). Abbreviations are as follows: DMSN, desmethylmensacarcin; IAA, indole-3-acetic acid.

We studied the distribution of previously characterized virulence determinants associated with potato common scab in the genomes of the six strains under study (Fig. 3). All strains lack the thaxtomin biosynthetic gene cluster, which is the main virulence determinant of most scab-causing species and strains [14, 51]. *Streptomyces* sp. nov. LBUM1542^T^ harbours the *sub1* gene, which encodes a suberinase [52, 53]. No other known virulence determinants were found in its genome apart from the IAA biosynthetic operon *iaaMH*. The genome of *Streptomyces* sp. nov. LBUM3076^T^ contains a putative BGC responsible for the production of DMSN. This BGC is also present in the genomes of *S. acidiscabies* ATCC 49003^T^ and *S. niveiscabiei* NRRL 24457^T^ (Fig. 3). This phytotoxic polyketide has been recently found to play an important role in the development of the potato common scab disease in strains lacking the thaxtomin BGC [54]. In addition, strain LBUM3076^T^ carries a putative BGC responsible for the biosynthesis of various rotihibin analogues that display herbicidal activities [55]. This BGC is absent from the genomes of the most closely related type strains (Fig. 3). This BGC was found in the genomes of other scab-causing strains that belong to the species *S. scabiei* and *S. stelliscabiei* [29, 55]. Lastly, strain LBUM3076^T^ is predicted to possess homologs of genes encoding the DNA-acting ADP-ribosyltransferase Scabin and the esterase EstA, two secreted proteins associated with potato common scab [52, 56].

### Morphology and physiology

**Fig. 4.**
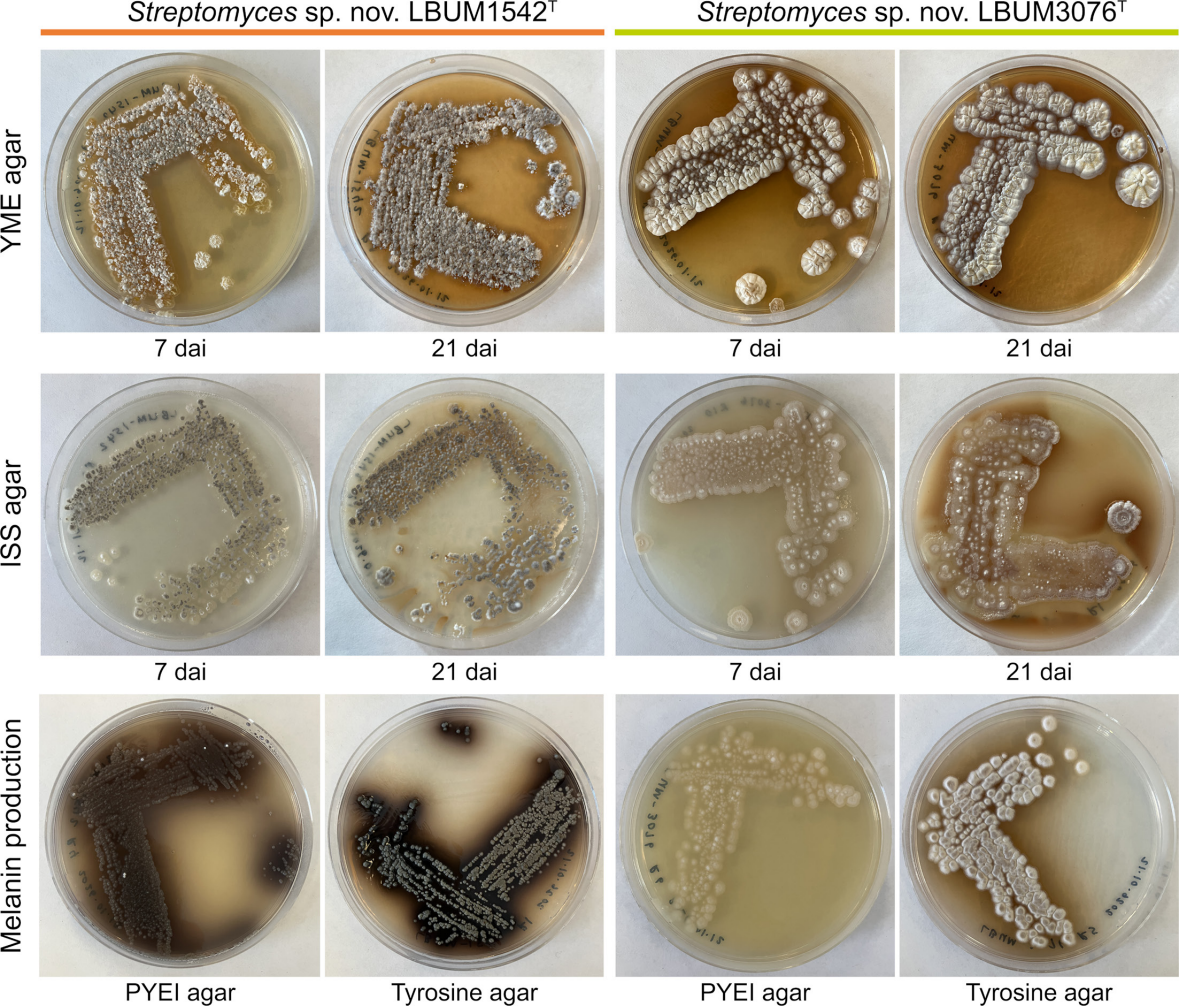
Morphological characteristics of strains LBUM1542^T^ and LBUM3076^T^. The strains were grown on YME agar, ISS agar, PYEI agar and tyrosine agar. Melanin production on PYEI agar and tyrosine agar was observed 4 days after inoculation.

The two proposed type strains were grown on various growth media to study colony and spore mass colours, as well as the presence of diffusible pigments. *Streptomyces* sp. nov. LBUM1542^T^ and *Streptomyces* sp. nov. LBUM3076^T^ grew well on YME agar, ISS agar and oat bran agar media. Moderate growth was observed on tryptone soy agar medium. Strain LBUM1542^T^ produced orange to light brown colonies on YME agar and off-white colonies on ISS agar (Fig. 4). A grey spore mass was produced on both media. Strain LBUM3076^T^ produced rust-brown colonies on YME agar, cream to light brown colonies on ISS agar and a greyish-white spore mass on both media (Fig. 4). Yellow droplets were produced by strain LBUM3076^T^ on YME agar. No diffusible pigments were produced by the strains on the tested media. Melanin production on PYEI agar and tyrosine agar was positive for LBUM1542^T^ and negative for LBUM3076^T^ (Fig. 4 and Table 2).

**Table 2.** Physiological characteristics of strains LBUM1542^T^ and LBUM3076^T^ and related *Streptomyces* type strains Data for the type strains of *S. rishiriensis*, *S. coriariae* and *S. zaehneri* were taken from Shelley *et al*. [27], Berckx *et al*. [62] and Nouioui *et al*. [63]. Data for *S. acidiscabies* DSM 41668^T^ were taken from Vitor *et al*. [26], except for the carbon source utilization data, which were obtained in this study.

Characteristic	*Streptomyces* sp. nov. LBUM1542^T^	*S. rishiriensis* NBRC 13407**^T^**	*S. coriariae* CMB-FB^T^	*S. zaehneri* DSM 40713**^T^**	*Streptomyces* sp. nov. LBUM3076^T^	*S. acidiscabies* DSM 41668**^T^**
**Melanin production**						
PYEI agar	+	nd	nd	nd	_	_
Tyrosine agar	+	nd	nd	nd	_	_
**Growth temperature range** (°C)						
4	_	nd	nd	_	_	nd
10	+	nd	nd	_	+	_
15	+	nd	_	+	+	_
20	+	nd	+	+	+	+
25	+	nd	+	+	+	+
28	+	nd	+	+	+	+
30	+	nd	+	+	+	+
37	+	nd	+	+	+	+
42	_	nd	_	_	_	nd
**Growth pH range**						
4	_	nd	nd	_	+	+
5	+	nd	_	+	+	+
6	+	nd	+	+	+	+
7	+	nd	+	+	+	+
8	+	nd	+	_	+	+
9	+	nd	_	_	+	+
10	_	nd	_	_	_	_
**NaCl tolerance range (%, w/v**)						
1	+	+	+	nd	+	+
2	+	+	+	nd	+	+
3	+	+	+	nd	+	+
4	+	_	+	nd	+	+
5	+	_	_	nd	+	+
6	+	_	_	nd	_	+
7	_	_	_	nd	_	_
**Carbon source utilization**						
d-Glucose	+	+	w	+	+	+
d-Fructose	+	+	_	+	+	+
d-Mannitol	_	w	+	_	_	+
d-Melibiose	_	+	nd	w	w	w
d-Raffinose	+	w	nd	_	_	_
d-Sucrose	+	+	+	+	+	+
d(+)-Trehalose	w	_	nd	w	w	+
*i*-Inositol	+	+	+	+	+	+
l-Arabinose	+	+	_	+	+	+

*Streptomyces* sp. nov. LBUM1542^T^ grew at temperatures between 10 and 37 °C (optimum 25–30 °C), at pH values ranging from 5 to 9 and in the presence of NaCl concentrations of up to 6.0% (w/v). While these values are similar to those obtained for closely related species, there were some differences (Table 2). For example, *S. rishiriensis* NBRC 13407^T^ only tolerated NaCl concentrations of up to 3.0% (w/v) [27]. *Streptomyces* sp. nov. LBUM3076^T^ grew at temperatures between 10 and 37 °C (optimum 25–30 °C), at pH values ranging from 4 to 9 and in the presence of NaCl concentrations of up to 5.0% (w/v). This strain shares the ability of *S. acidiscabies* ATCC 49003^T^ to grow at a pH of 4 [16], but the latter can tolerate NaCl concentrations of up to 6% (w/v) [26].

The ability of the strains under study to utilize various carbon sources was tested and compared with several closely related type strains (Table 2). Strain LBUM1542^T^ was able to use d-glucose, d-fructose, raffinose, sucrose, *i*-inositol and l-arabinose as sole carbon sources, but not d-mannitol or melibiose. This strain differs from its closest related type strains in the utilization of at least one carbon source; for example, *S. rishiriensis* NBRC 13407^T^ can utilize melibiose. Strain LBUM3076^T^ utilized d-glucose, d-fructose, sucrose, *i*-inositol and l-arabinose, but not d-mannitol or raffinose. By contrast, its most closely related type strain, *S. acidiscabies* ATCC 49003^T^, is able to use raffinose as the sole source of carbon (Table 2).

Lastly, various biochemical tests were performed to characterize the two representative type strains. Both strains are Gram-positive, aerobic and non-motile. Catalase activity is positive, but oxidase activity and nitrate reduction are negative. Gelatin liquefaction is weakly positive for both strains. Starch hydrolysis is weakly positive for strain LBUM1542^T^ and positive for strain LBUM3076^T^. Casein hydrolysis is negative for LBUM1542^T^ and weakly positive for LBUM3076^T^.

### Carbohydrate-active enzyme repertoire

**Fig. 5.**
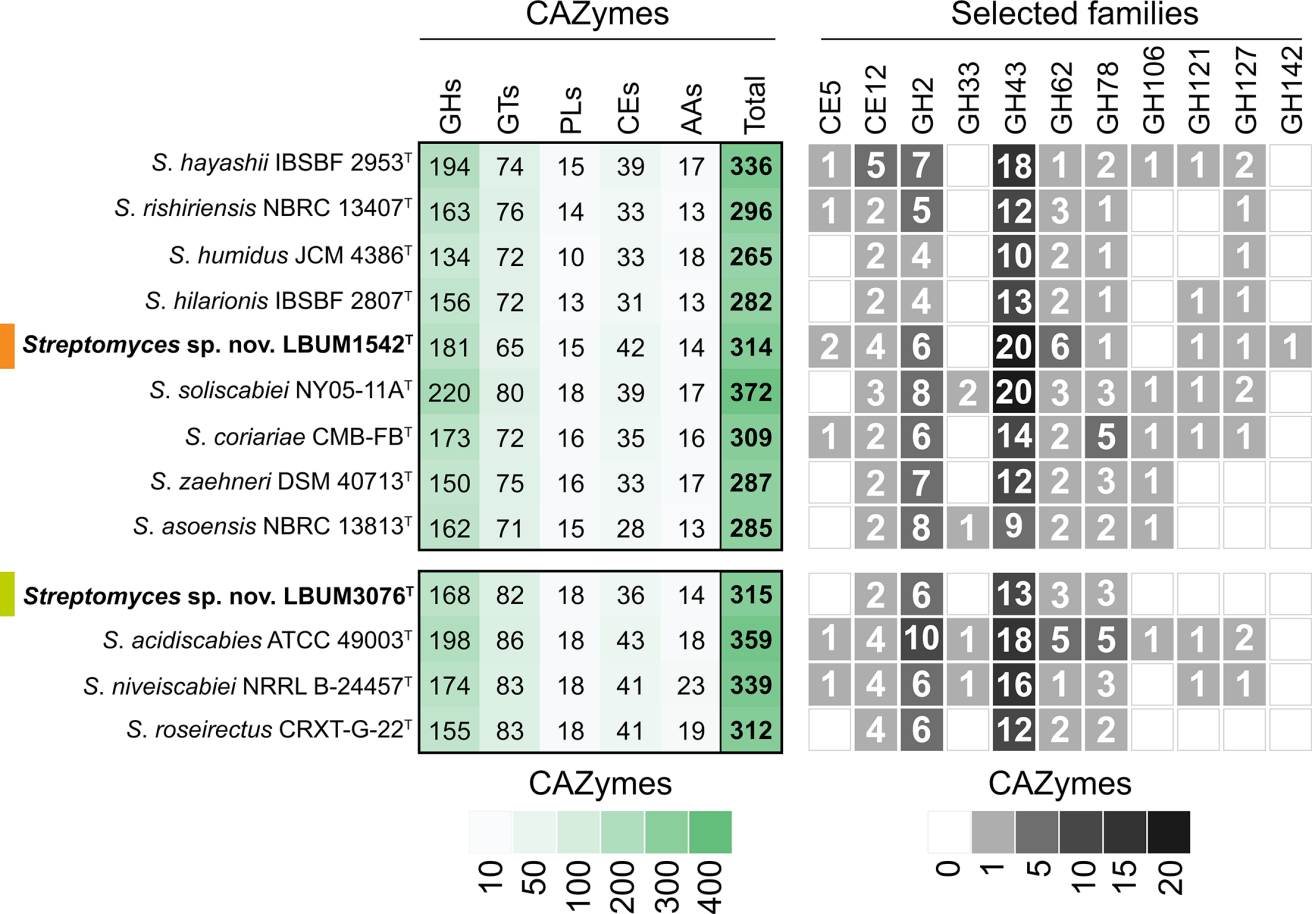
Putative CAZymes encoded in the genomes of the strains under study. These enzymes can be classified into five classes, namely GHs, GTs, PLs, CEs and auxiliary activities (AAs), and into numerous families, such as the glycoside hydrolase family 62 (GH62). For example, strain LBUM1542^T^ possesses six genes encoding putative CAZymes that belong to the GH62 family.

An *in silico* analysis was performed to identify genes encoding putative CAZymes in the genomes of the two proposed type strains and in the genomes of the most closely related type strains. *Streptomyces* sp. nov. LBUM1542^T^ possesses 314 putative CAZyme-encoding genes, including 181 glycoside hydrolases (GHs), 65 glycosyltransferases (GTs), 15 polysaccharide lyases (PLs) and 42 carbohydrate esterases (CEs). The CAZyme repertoire of strain LBUM1542^T^ is smaller than those of the scab-causing strains *S. soliscabiei* NY05-11A^T^ and *S. hayashii* IBSBF 2953^T^. One of the most striking features of *Streptomyces* sp. nov. LBUM1542^T^ that differentiates this strain from the most closely related type strains is the abundance of putative arabinofuranosidases encoded in its genome. Indeed, this strain harbours 20 and 6 genes encoding putative enzymes that belong to the GH43 and GH62 families, respectively (Fig. 5, Tables S3 and S4). Many of them are predicted to function as α-l-arabinofuranosidases. In addition, strain LBUM1542^T^ possesses in its genome a gene encoding a putative *β*-l-arabinofuranosidase from the GH142 family, which is absent from other strains (Fig. 5). *Streptomyces* sp. nov. LBUM3076^T^ harbours 315 putative CAZyme-encoding genes, including 168 GHs, 82 GTs, 18 PLs and 36 CEs. The CAZyme repertoire of strain LBUM3076^T^ is more limited than that of *S. acidiscabies* ATCC 49003^T^ (359 putative CAZymes). This difference is even more pronounced when comparing genes encoding putative GHs (Fig. 5). Strain LBUM3076^T^ lacks genes encoding putative CAZymes belonging to the CE05, GT8, GH33, GH106, GH121 and GH127 families, which are present in the genome of *S. acidiscabies* ATCC 49003^T^. These results indicate that *Streptomyces* sp. nov. LBUM3076^T^ and *S. acidiscabies* ATCC 49003^T^ differ significantly in their carbohydrate degradation capacities, supporting their classification as distinct species.

### Genome-based prediction of chemotaxonomy

An *in silico* approach was used to predict the presence of several chemotaxonomic markers in *Streptomyces* sp. nov. LBUM1542^T^ and *Streptomyces* sp. nov. LBUM3076^T^. This genome-based approach was previously used in the formal description of several *Streptomyces* species [24, 25, 27]. The presence/absence of genes encoding enzymes involved in several biosynthetic pathways (fatty acids, mycolic acids, menaquinones and 2,6-diaminopimelic acid) is presented in Table S5. *Streptomyces* sp. nov. LBUM1542^T^ and *Streptomyces* sp. nov. LBUM3076^T^ are predicted to synthesize fatty acids via the type II fatty acid synthase (FAS) pathway. The strains possess genes encoding most of the enzymes involved in the type II FAS pathway, only lacking *kasB* and *hadA* orthologs. They both lack type I FAS-encoding genes. In addition, the two strains lack many genes involved in the biosynthesis of mycolic acid, including *cmaA1*, *cmaA2*, *umaA1*, *pcaA*, *mmaA1*, *mmaA2*, *mmaA3* and *mmaA4*. These eight genes encode key enzymes involved in the modification of the meromycolic chain [57]. Therefore, it is unlikely that the two strains produce mycolic acids. Both strains are predicted to produce menaquinone via the futalosine-dependent menaquinone biosynthesis pathway. Indeed, the two strains lack most genes encoding enzymes of the classical menaquinone biosynthesis pathway, but harbour genes predicted to encode the enzymes of the menaquinone biosynthetic pathway discovered in *Streptomyces coelicolor* [58]. In addition, they both lack a homologue of the *menJ* gene, which encodes a menaquinone reductase [59]. This enzyme is responsible for the conversion of MK-9 to MK-9(II–H_2_), and, therefore, the two strains do not produce MK-9(II–H_2_) as the main type of menaquinone. Finally, both strains harbour a homologue of the *murE* gene involved in the production of 2,6-diaminopimelic acid (DAP), suggesting they produce isomers of DAP. These results are in agreement with the chemotaxonomic markers commonly reported for *Streptomyces* spp. [60, 61].

It appears evident from the data presented above that strains LBUM1542^T^ and LBUM3076^T^ represent novel species within the genus *Streptomyces*, for which the names *Streptomyces borealis* sp. nov. and *Streptomyces saintjeani* sp. nov. are proposed.

## Description of *Streptomyces borealis* sp. nov.

*Streptomyces borealis* (bo.re.a’lis. L. masc. adj. *borealis*, pertaining to the North, boreal)

Aerobic, Gram-positive, non-motile bacteria in the phylum *Actinomycetota*. Colonies are orange to light brown on yeast malt extract agar and off-white on inorganic salts-starch agar, with grey spore masses produced on both media. Grows well on yeast malt extract agar, inorganic salts-starch agar and oat bran agar. Moderate growth was observed on tryptone soy agar. Melanin is produced on tyrosine agar and peptone-yeast extract iron agar. Growth occurs at temperatures between 10 and 37 °C (optimum 25–30 °C), at pH values ranging from 5 to 9 and in the presence of up to 6.0% (w/v) NaCl. Utilizes d-glucose, d-fructose, d-raffinose, d-sucrose, *i*-inositol and l-arabinose as sole carbon sources, but not d-mannitol and d-melibiose. Positive for catalase, but negative for oxidase. Gelatin liquefaction and starch hydrolysis are weakly positive. Nitrate reduction and casein hydrolysis are negative.

The type strain, LBUM1542^T^ (=NCPPB 4834^T^=NRRL B-65796^T^), was isolated from a common scab lesion on a potato tuber harvested from a potato field in Dolbeau-Mistassini, Quebec, Canada. The genome size of the type strain is 9.87 Mb, and the DNA G+C content is 71.4 mol%. The GenBank accession numbers for the 16S rRNA gene and the whole-genome shotgun sequence of the type strain are PX939437 and JARUNH000000000.1, respectively.

## Description of *Streptomyces saintjeani* sp. nov.

*Streptomyces saintjeani* (saint.jean’i. N.L. gen. n. *saintjeani*, named after Lac Saint-Jean, Quebec, Canada, near the site where the type strain was isolated)

Aerobic, Gram-positive, non-motile bacteria in the phylum *Actinomycetota*. Colonies are rust-brown on yeast malt extract agar and cream to light brown on inorganic salts-starch agar, with greyish-white spore masses produced on both media. Yellow droplets are observed on yeast malt extract agar. Grows well on yeast malt extract agar, inorganic salts-starch agar and oat bran agar. Moderate growth was observed on tryptone soy agar. Melanin is not produced on tyrosine agar and peptone–yeast extract iron agar. Growth occurs at temperatures between 10 and 37 °C (optimum 25–30 °C), at pH values ranging from 4 to 9 (optimum 6–9) and in the presence of up to 5.0% (w/v) NaCl. Utilizes d-glucose, d-fructose, d-sucrose, *i*-inositol and l-arabinose as sole carbon sources, but not d-mannitol and d-raffinose. Positive for catalase, but negative for oxidase. Starch hydrolysis is positive. Gelatin liquefaction and casein hydrolysis are weakly positive. Nitrate reduction is negative.

The type strain, LBUM3076^T^ (=NCPPB 4835^T^=NRRL B-65797^T^), was isolated from a common scab lesion on a potato tuber harvested from a potato field in Dolbeau-Mistassini, Quebec, Canada. The genome size of the type strain is 9.83 Mb, and the DNA G+C content is 71.0 mol%. The GenBank accession numbers for the 16S rRNA gene and the complete genome sequence of the type strain are PX939436 and CP189624.1, respectively.

## Supplementary material

10.1099/ijsem.0.007254 Supplementary Material 1.

10.1099/ijsem.0.007254 Supplementary Material 2.

## Abbreviations

ANIb: average nucleotide identity based on blast+
ANIm: average nucleotide identity based on MUMmer
BGCs: biosynthetic gene clusters
CAZymes: carbohydrate-active enzymes
CEs: carbohydrate esterases
DAP: 2,6-diaminopimelic acid
dDDH: digital DNA–DNA hybridization
FAS: fatty acid synthase
GHs: glycoside hydrolases
GTR: generalized time-reversible
GTs: glycosyltransferases
ISS: inorganic salts-starch
MLSA: multilocus sequence analysis
OGRIs: overall genome relatedness indices
PLs: polysaccharide lyases
PYEI: peptone–yeast extract iron
TSB: tryptic soy broth
YME: yeast–malt extract

## References

[1] Norte DM, Avitia-Dominguez LA, Rozen DE (2025). Evolution and ecology of *Streptomyces*. Annu Rev Microbiol.

[2] Komaki H (2023). Recent progress of reclassification of the genus *Streptomyces*. Microorganisms.

[3] Waksman SA (1953). Streptomycin: background, isolation, properties, and utilization. Science.

[4] Watve MG, Tickoo R, Jog MM, Bhole BD (2001). How many antibiotics are produced by the genus *Streptomyces*?. Arch Microbiol.

[5] Donald L, Pipite A, Subramani R, Owen J, Keyzers RA (2022). *Streptomyces*: still the biggest producer of new natural secondary metabolites, a current perspective. Microbiol Res.

[6] Parte AC (2014). LPSN--list of prokaryotic names with standing in nomenclature. Nucleic Acids Res.

[7] Freese HM, Meier-Kolthoff JP, Sardà Carbasse J, Afolayan AO, Göker M (2026). TYGS and LPSN in 2025: a Global Core Biodata Resource for genome-based classification and nomenclature of prokaryotes within DSMZ Digital Diversity. Nucleic Acids Res.

[8] Goyer C, Beaulieu C (1997). Host range of streptomycete strains causing common scab. *Plant Dis*.

[9] Clarke CR, Kramer CG, Kotha RR, Luthria DL (2022). The phytotoxin thaxtomin A is the primary virulence determinant for scab disease of beet, carrot, and radish caused by *Streptomyces scabiei*. Phytopathology.

[10] Loria R, Bukhalid RA, Fry BA, King RR (1997). Plant pathogenicity in the genus *Streptomyces*. *Plant Dis*.

[11] Dees MW, Wanner LA (2012). In search of better management of potato common scab. *Potato Res*.

[12] Loria R, Coombs J, Yoshida M, Kers J, Bukhalid R (2003). A paucity of bacterial root diseases: *Streptomyces* succeeds where others fail. Physiol Mol Plant Pathol.

[13] Li Y, Liu J, Díaz-Cruz G, Cheng Z, Bignell DRD (2019). Virulence mechanisms of plant-pathogenic *Streptomyces* species: an updated review. *Microbiology*.

[14] Loria R, Bignell DRD, Moll S, Huguet-Tapia JC, Joshi MV (2008). Thaxtomin biosynthesis: the path to plant pathogenicity in the genus *Streptomyces*. Antonie van Leeuwenhoek.

[15] Lambert DH, Loria R (1989). *Streptomyces scabies* sp. nov., nom. rev. Int J Syst Evol Microbiol.

[16] Lambert DH, Loria R (1989). *Streptomyces acidiscabies* sp. nov. Int J Syst Bacteriol.

[17] Wanner LA (2009). A patchwork of *Streptomyces* species isolated from potato common scab lesions in North America. Am J Pot Res.

[18] Tashiro N, Manabe K, Saito A, Miyashita K (2012). Identification of potato scab-causing *Streptomyces* sp. occurring in strongly acidic soils in Saga Prefecture in Japan. J Gen Plant Pathol.

[19] Uysal N, Bozkurt A, Elçi E (2025). Isolation and characterization of plant‐pathogenic *Streptomyces* species associated with potato common scab disease in Türkiye. Plant Pathol.

[20] Goyer C, Faucher E, Beaulieu C (1996). *Streptomyces caviscabies* sp. nov., from deeppitted lesions in potatoes in Québec, Canada. Int J Syst Bacteriol.

[21] Miyajima K, Tanaka F, Takeuchi T, Kuninaga S (1998). *Streptomyces turgidiscabies* sp. nov. Int J Syst Bacteriol.

[22] Bouchek-Mechiche K, Gardan L, Normand P, Jouan B (2000). DNA relatedness among strains of *Streptomyces* pathogenic to potato in France: description of three new species, *S. europaeiscabiei* sp. nov. and *S. stelliscabiei* sp. nov. associated with common scab, and *S. reticuliscabiei* sp. nov. associated with netted scab. Int J Syst Evol Microbiol.

[23] Park DH, Kim JS, Kwon SW, Wilson C, Yu YM (2003). *Streptomyces luridiscabiei* sp. nov., *Streptomyces puniciscabiei* sp. nov. and *Streptomyces niveiscabiei* sp. nov., which cause potato common scab disease in Korea. Int J Syst Evol Microbiol.

[24] Nguyen HP, Weisberg AJ, Chang JH, Clarke CR (2022). *Streptomyces caniscabiei* sp. nov., which causes potato common scab and is distributed across the world. Int J Syst Evol Microbiol.

[25] Nguyen HP, Shelley BA, Mowery J, Clarke CR (2022). Description of *Streptomyces griseiscabiei* sp. nov. and reassignment of *Streptomyces* sp. strain NRRL B-16521 to *Streptomyces acidiscabies*. Int J Syst Evol Microbiol.

[26] Vitor L, Amaral DT, Corrêa DBA, Ferreira-Tonin M, Lucon ET (2023). *Streptomyces hilarionis* sp. nov. and *Streptomyces hayashii* sp. nov., two new strains associated with potato scab in Brazil. Int J Syst Evol Microbiol.

[27] Shelley BA, Clarke CR (2025). Description of *Streptomyces soliscabiei* sp. nov. and *Streptomyces echiniscabiei* sp. nov., which cause common scab disease on *Solanum tuberosum*. Int J Syst Evol Microbiol.

[28] Hudec C, Novinscak A, Filion M (2021). Diversity and virulence of *Streptomyces* spp. causing potato common scab in Prince Edward Island, Canada. Phytopathology.

[29] Biessy A, Cadieux M, Ciotola M, St-Onge R, Blom J (2024). Virulence determinants are unevenly distributed within *Streptomyces* species and strains causing potato common scab in the province of Quebec, Canada. *Plant Dis*.

[30] Flores‐González R, Velasco I, Montes F (2008). Detection and characterization of *Streptomyces* causing potato common scab in Western Europe. Plant Pathol.

[31] Leiminger J, Frank M, Wenk C, Poschenrieder G, Kellermann A (2013). Distribution and characterization of *Streptomyces* species causing potato common scab in Germany. Plant Pathol.

[32] Lapaz MI, Huguet-Tapia JC, Siri MI, Verdier E, Loria R (2017). Genotypic and phenotypic characterization of *Streptomyces* species causing potato common scab in Uruguay. *Plant Dis*.

[33] Conn KL, Leci E, Kritzman G, Lazarovits G (1998). A quantitative method for determining soil populations of *Streptomyces* and differentiating potential potato scab-inducing strains. *Plant Dis*.

[34] Kolmogorov M, Yuan J, Lin Y, Pevzner PA (2019). Assembly of long, error-prone reads using repeat graphs. Nat Biotechnol.

[35] Tatusova T, DiCuccio M, Badretdin A, Chetvernin V, Nawrocki EP (2016). NCBI prokaryotic genome annotation pipeline. Nucleic Acids Res.

[36] Medema MH, Blin K, Cimermancic P, de Jager V, Zakrzewski P (2011). antiSMASH: rapid identification, annotation and analysis of secondary metabolite biosynthesis gene clusters in bacterial and fungal genome sequences. Nucleic Acids Res.

[37] Blin K, Shaw S, Vader L, Szenei J, Reitz ZL (2025). antiSMASH 8.0: extended gene cluster detection capabilities and analyses of chemistry, enzymology, and regulation. Nucleic Acids Res.

[38] Yoon S-H, Ha S-M, Kwon S, Lim J, Kim Y (2017). Introducing EzBioCloud: a taxonomically united database of 16S rRNA gene sequences and whole-genome assemblies. Int J Syst Evol Microbiol.

[39] Sievers F, Wilm A, Dineen D, Gibson TJ, Karplus K (2011). Fast, scalable generation of high-quality protein multiple sequence alignments using Clustal Omega. Mol Syst Biol.

[40] Price MN, Dehal PS, Arkin AP (2009). FastTree: computing large minimum evolution trees with profiles instead of a distance matrix. Mol Biol Evol.

[41] Price MN, Dehal PS, Arkin AP (2010). FastTree 2--approximately maximum-likelihood trees for large alignments. PLoS One.

[42] Shimodaira H, Hasegawa M (1999). Multiple comparisons of log-likelihoods with applications to phylogenetic inference. Mol Biol Evol.

[43] Meier-Kolthoff JP, Auch AF, Klenk H-P, Göker M (2013). Genome sequence-based species delimitation with confidence intervals and improved distance functions. *BMC Bioinformatics*.

[44] Richter M, Rosselló-Móra R, Oliver Glöckner F, Peplies J (2016). JSpeciesWS: a web server for prokaryotic species circumscription based on pairwise genome comparison. Bioinformatics.

[45] Shirling EB, Gottlieb D (1966). Methods for characterization of *Streptomyces* species. Int J Syst Bacteriol.

[46] Goyer C, Vachon J, Beaulieu C (1998). Pathogenicity of *Streptomyces* scabies mutants altered in thaxtomin A production. Phytopathology.

[47] Yin Y, Mao X, Yang J, Chen X, Mao F (2012). dbCAN: a web resource for automated carbohydrate-active enzyme annotation. Nucleic Acids Res.

[48] Zheng J, Ge Q, Yan Y, Zhang X, Huang L (2023). dbCAN3: automated carbohydrate-active enzyme and substrate annotation. Nucleic Acids Res.

[49] Ciufo S, Kannan S, Sharma S, Badretdin A, Clark K (2018). Using average nucleotide identity to improve taxonomic assignments in prokaryotic genomes at the NCBI. Int J Syst Evol Microbiol.

[50] Hu S, Li K, Zhang Y, Wang Y, Fu L (2022). New insights into the threshold values of multi-locus sequence analysis, average nucleotide identity and digital DNA–DNA hybridization in delineating *Streptomyces* species. Front Microbiol.

[51] King RR, Calhoun LA (2009). The thaxtomin phytotoxins: sources, synthesis, biosynthesis, biotransformation and biological activity. Phytochemistry.

[52] Komeil D, Simao-Beaunoir A-M, Beaulieu C (2013). Detection of potential suberinase-encoding genes in *Streptomyces scabiei* strains and other actinobacteria. Can J Microbiol.

[53] Jabloune R, Khalil M, Ben Moussa IE, Simao-Beaunoir A-M, Lerat S (2020). Enzymatic degradation of p-nitrophenyl esters, polyethylene terephthalate, cutin, and suberin by Sub1, a suberinase encoded by the plant pathogen *Streptomyces scabies*. Microb Environ.

[54] Lapaz MI, Zeballos-Gorón S, Croce V, Huguet-Tapía JC, Pérez-Baldassari M (2026). Identification of the biosynthetic gene cluster for desmethylmensacarcin, a phytotoxic polyketide produced by *Streptomyces niveiscabiei* ST1015. Physiol Mol Plant Pathol.

[55] Planckaert S, Deflandre B, de Vries A-M, Ameye M, Martins JC (2021). Identification of novel rotihibin analogues in *Streptomyces scabies*, including discovery of its biosynthetic gene cluster. Microbiol Spectr.

[56] Lyons B, Ravulapalli R, Lanoue J, Lugo MR, Dutta D (2016). Scabin, a novel DNA-acting ADP-ribosyltransferase from *Streptomyces scabies*. J Biol Chem.

[57] Marrakchi H, Lanéelle M-A, Daffé M (2014). Mycolic acids: structures, biosynthesis, and beyond. Chem Biol.

[58] Hiratsuka T, Furihata K, Ishikawa J, Yamashita H, Itoh N (2008). An alternative menaquinone biosynthetic pathway operating in microorganisms. Science.

[59] Upadhyay A, Fontes FL, Gonzalez-Juarrero M, McNeil MR, Crans DC (2015). Partial saturation of menaquinone in *Mycobacterium tuberculosis*: function and essentiality of a novel reductase, MenJ. ACS Cent Sci.

[60] Gago G, Diacovich L, Arabolaza A, Tsai S-C, Gramajo H (2011). Fatty acid biosynthesis in actinomycetes. FEMS Microbiol Rev.

[61] Kämpfer P, Dworkin M, Falkow S, Rosenberg E, Schleifer K-H, Stackebrandt E (2006). The Prokaryotes.

[62] Berckx F, Bandong CM, Wibberg D, Kalinowski J, Willemse J (2022). *Streptomyces coriariae* sp. nov., a novel streptomycete isolated from actinorhizal nodules of *Coriaria intermedia*. Int J Syst Evol Microbiol.

[63] Nouioui I, Zimmermann A, Hennrich O, Xia S, Rössler O (2024). Challenging old microbiological treasures for natural compound biosynthesis capacity. Front Bioeng Biotechnol.

