# Supplementary material for: Description of Streptomyces borealis sp. nov. and Streptomyces saintjeani sp. nov., associated with potato common scab
Source: Int J Syst Evol Microbiol. 2026 Jul 27;76(7):007254. doi: 10.1099/ijsem.0.007254 (PMC13403950; doi:10.1099/ijsem.0.007254)
Supplement: Supplementary Material 1. [file ijsem-76-07254-s001.pdf]

SUPPLEMENTARY FIGURES

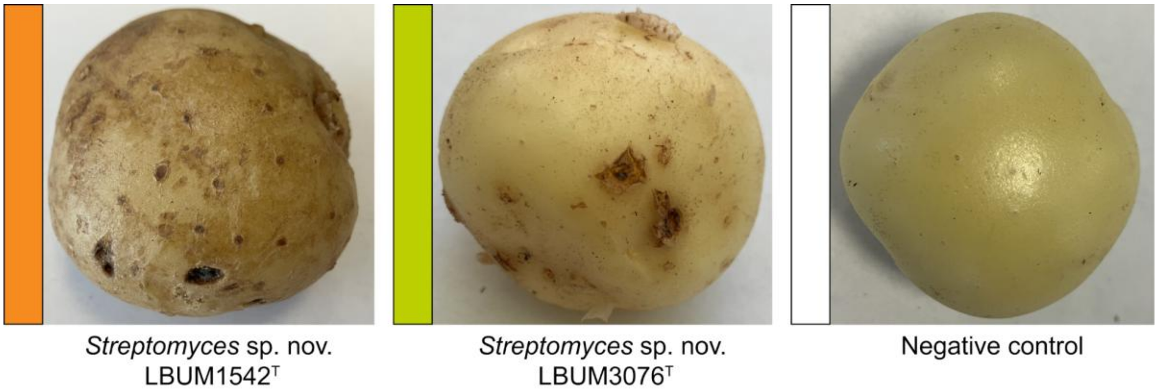

Figure S1. Representative symptoms caused by the strains belonging to the species-level groups described in this study.

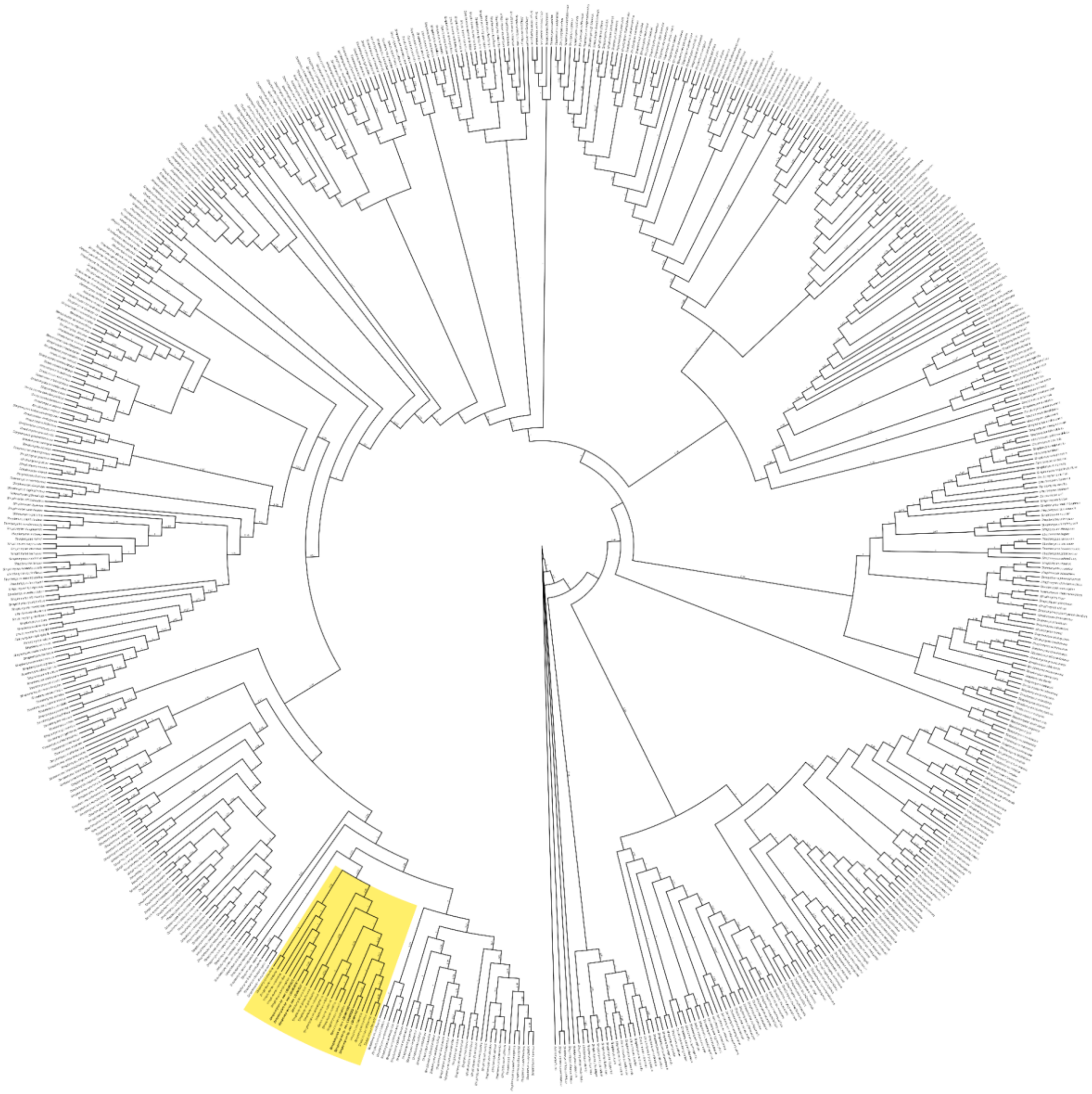

Figure S2. Multilocus sequence analysis within the genus *Streptomyces*. The complete nucleotide sequences of five housekeeping genes (*atpD*, *gyrB*, *recA*, *rpoB*, and *trpB*) were used to generate a phylogenetic tree with the approximate maximum-likelihood algorithm FastTree 2.1 and the GTR model. More than 650 *Streptomyces* type strains are included in the tree. The six strains under study clustered within the yellow clade, which notably includes the scab-causing pathogens *S. acidiscabies*, *S. niveiscabiei*, and *S. soliscabiei*. Shimodaira Hasegawa support values are displayed at the nodes and *Actinomyces bovis* NCTC 11535^{T} was used as an outgroup.

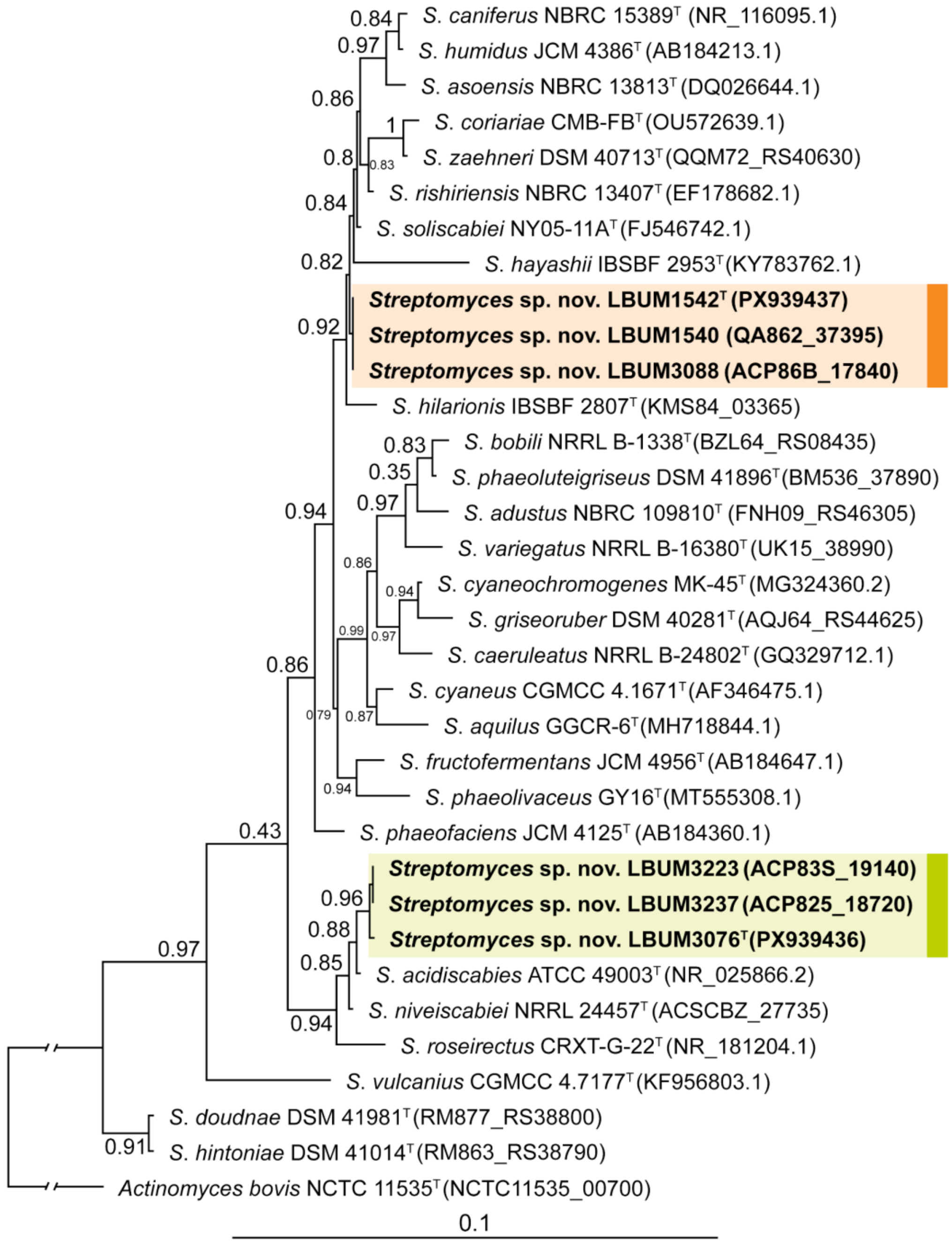

Figure S3. Phylogenetic tree based on phylogenetic analysis of the 16S rRNA gene. The 16S rDNA nucleotide sequences were retrieved from GenBank and used to generate a maximum-likelihood tree using FastTree 2.1 and the GTR model. The six strains under study are highlighted in colors. Shimodaira Hasegawa support values are displayed at the nodes and *Actinomyces bovis* NCTC 11535^{T} was used as an outgroup.
